# Plant-Derived Agents for Counteracting Cisplatin-Induced Nephrotoxicity

**DOI:** 10.1155/2016/4320374

**Published:** 2016-09-27

**Authors:** Shreesh Ojha, Balaji Venkataraman, Amani Kurdi, Eglal Mahgoub, Bassem Sadek, Mohanraj Rajesh

**Affiliations:** ^1^Department of Pharmacology and Therapeutics, College of Medicine and Health Sciences, United Arab Emirates University, Al Ain 17666, UAE; ^2^Department of Pharmacology and Therapeutics, Beirut Arab University, Beirut, Lebanon

## Abstract

Cisplatin (CSP) is a chemotherapeutic agent commonly used to treat a variety of malignancies. The major setback with CSP treatment is that its clinical efficacy is compromised by its induction of organ toxicity, particular to the kidneys and ears. Despite the significant strides that have been made in understanding the mechanisms underlying CSP-induced renal toxicity, advances in developing renoprotective strategies are still lacking. In addition, the renoprotective approaches described in the literature reveal partial amelioration of CSP-induced renal toxicity, stressing the need to develop potent combinatorial/synergistic agents for the mitigation of renal toxicity. However, the ideal renoprotective adjuvant should not interfere with the anticancer efficacy of CSP. In this review, we have discussed the progress made in utilizing plant-derived agents (phytochemicals) to combat CSP-induced nephrotoxicity in preclinical studies. Furthermore, we have also presented strategies to utilize phytochemicals as prototypes for the development of novel renoprotective agents for counteracting chemotherapy-induced renal damage.

## 1. Introduction

Cisplatin (CSP), chemically known as* cis*-diamminedichloroplatinum-II, is an anticancer agent used in the treatment of testicular, head and neck, ovarian, cervical, and non-small-cell lung cancers [1]. The major issues limiting the clinical use of CSP are its tendency to induce profound nephrotoxicity and ototoxicity [1]. The first occurrence of nephrotoxicity was documented in the clinical trial that evaluated the anticancer effects of CSP. It is estimated that 30% of patients treated with CSP could exhibit elevated serum creatinine levels and reduced glomerular filtration rate, reflecting the development of nephrotoxicity. In addition, these symptoms could occur as early as 10 days after the initiation of CSP chemotherapy. Moreover, nephrotoxicity is considered a determinant side effect of the use of anticancer medications. It is pertinent to note that approximately 50–60% of patients undergoing cancer chemotherapy acquire nosocomial acute kidney injury, which is associated with increased morbidity and mortality rates [1, 2].

The pathophysiological mechanisms purported to underlie CSP-induced nephrotoxicity have been extensively studied, and several hypotheses have been forwarded. To date, oxidative stress, inflammation, and apoptosis pathways have been widely considered as key pathomechanisms involved in the CSP-induced nephrotoxicity [3]. The identified scenario is that the accumulation of CSP in renal tissues results in massive oxidative stress that causes inflammatory damage to the tubular epithelium, which spreads to the renal microvasculature, impedes the blood flow by evoking ischemic injury, and decreases the glomerular filtration rate. These phenotypic events culminate in acute renal failure. To circumvent the CSP-induced nephrotoxicity, several analogs have been developed, which are expected to be less nephrotoxic. In addition, several clinical trials have examined the efficacy of mannitol and furosemide (osmotic and loop diuretics, resp.) in reducing the renal retention of CSP and, thereby, minimizing the noxious effects on naïve tissue [4]. However, this approach has met with limited clinical success; while the induced nephrotoxicity has been milder, it has not been completely averted. Therefore, there is an urgent need to develop agents that confer renoprotection without compromising the anticancer activity of CSP [1, 5].

## 2. Phytochemicals as Leads for Attenuating CSP-Induced Nephrotoxicity

Phytochemicals are compounds that are distributed in various plant tissues and are responsible for imparting characteristics such as color and smell but do not possess nutritional value. Importantly, phytochemicals have been used in traditional medicine for several centuries for treating various ailments. There is considerable evidence from* in vitro* preclinical studies that phytochemicals extracted from various plant sources may retard tumor growth and elicit antioxidant and anti-inflammatory effects [6]. Most importantly, the anticancer agent Taxol (paclitaxel) is a phytochemical that was originally identified, extracted, and purified from the bark of the Pacific yew tree (*Taxus brevifolia*) [7]. Currently, most developed drugs are not from plants but are rather chemically synthesized. Recently there is a renewed interest in tapping into the potential of medicinal plants in drug discovery, since phytochemicals are chemically diverse in nature and a considerable receptacle of pharmacophores. This enthusiasm has led to significant research strides in the identification of several potential phytochemicals that are being investigated for their renoprotective actions in preclinical studies.

Extensive investigations over the past decade have provided significant insights into the pathophysiology of CSP-induced nephrotoxicity. A plethora of biochemical pathways and mechanisms have been purported to mediate CSP-elicited nephrotoxicity, including those involved in oxidative/nitrative stress, mitochondrial malfunction, inflammation, and cell death [reviewed in [8–10]]. Recently, the involvement of endocannabinoid system has been implicated in the pathogenesis of CSP-induced nephrotoxicity [11, 12].

**Figure 1:**
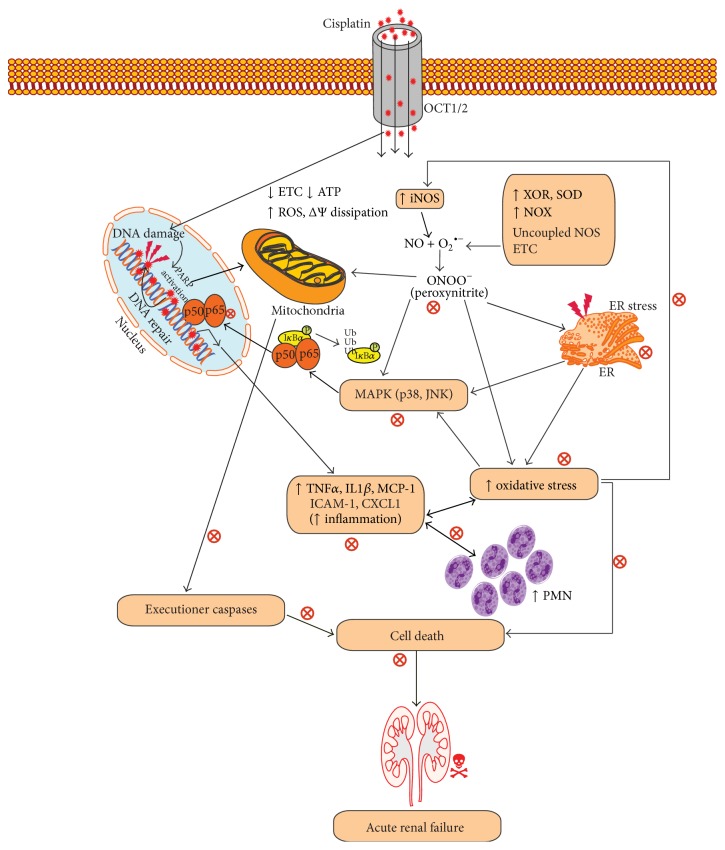
Scheme showing various pathways mediating cisplatin- (CSP-) induced nephrotoxicity and mitigation of this cascade by phytochemicals.

In this context, we have discussed the developments made with the use of phytochemicals to attenuate the development CSP-induced nephrotoxicity in experimental models. The summary of the effects of phytochemicals in preclinical or* ex vivo* studies or both is provided in Table 1. The chemical structures of phytochemicals that have been tested for potential renoprotective actions against CSP-induced renal toxicity are presented in Table 2. Next, various biochemical pathways recruited by CSP in eliciting renal toxicity and the attenuation of these effects by phytochemicals are illustrated in Figure 1. Furthermore, in the following section, we systematically discussed the effects of various phytochemicals investigated for their potential renoprotection against CSP-induced nephrotoxicity.

### 2.1. 23-Hydroxytormentic Acid (23-HTA) and Niga-ichigoside F_1_ (NIF_1_)

23-Hydroxytormentic acid (23-HTA), an aglycone of the triterpenoid glycoside niga-ichigoside F_1_ (NIF_1_), has been isolated from the unripe fruit of* Rubus coreanus*, a perennial shrub found in southern parts of Korea [13]. Kim et al. [13] and Sohn et al. [14] have demonstrated that 23-HTA and NIF_1_ attenuated CSP-induced nephrotoxicity by mitigating oxidative stress and inflammation in renal tissues. However, further mechanistic studies are required to confirm their renoprotective effects against CSP-induced renal toxicity.

### 2.2. 6-Gingerol

6-Gingerol is a pungent ingredient of ginger (*Zingiber officinale*), which has demonstrated anti-inflammatory, analgesic, antipyretic, antitumor, and antiproliferative properties [15, 16]. Kuhad et al. [17] reported that gingerol inhibited CSP-induced nephrotoxicity by suppressing oxidative stress. Similarly, another study reported that gingerol elicited renoprotective action by mitigating renal oxidative stress and inflammation [18]. However, further studies are warranted to delineate the precise molecular mechanisms of their renoprotective actions.

### 2.3. 6-Hydroxy-1-methylindole-3-acetonitrile (6-HMA)

6-HMA is a phytochemical present in* Brassica rapa* roots. In traditional medicine,* B. rapa* has been used to treat a variety of conditions such as hepatitis, jaundice, furuncle, and sore throats [19]. 6-HMA has been demonstrated to improve renal function, augment endogenous antioxidant defenses, and protect kidneys from the noxious effects of CSP. Further, 6-HMA also inhibited CSP-induced death of LLC-PK1 cells (renal proximal tubular epithelial cells derived from porcine kidneys) [19].

### 2.4. *β*-Caryophyllene (BCP)

*β*-Caryophyllene (BCP) is a natural sesquiterpene found in several essential oils of spices such as cinnamon, oregano, black pepper, basil, cloves, and other condiments [20]. BCP has been shown to elicit anti-inflammatory [20] and antioxidant effects [21, 22]. Horváth et al. [23] demonstrated that BCP attenuated CSP-induced nephrotoxicity by decreasing oxidative/nitrative stress, inflammation, and cell death pathway activation. Further, mechanistic studies revealed that the renoprotective actions of BCP against CSP-induced renal toxicity were mediated via activation of cannabinoid receptor-2 (CB_2_). It is pertinent to note that previous studies have also demonstrated the renoprotective role of CB_2_ receptor activation [24]. In addition, several studies have documented the anti-inflammatory phenotype induced by CB_2_ receptors activation in preclinical studies [25]. Considering the good safety and tolerability profile of BCP in human subjects, this has excellent prospects for further pharmaceutical development as a renoprotective agent.

### 2.5. Berberine

Berberine, an isoquinoline alkaloid present in the rhizome, root, and stem bark of several plant species, is especially highly concentrated in berries (*Berberis vulgaris*) [26]. Berberine has been documented to possess antioxidant, anti-inflammatory, and anticancer activities [26]. Berberine inhibited CSP-induced nephrotoxicity by reducing oxidative stress/nitrative stress, nuclear factor kappa-light-chain-enhancer of activated B-cells (NF*κ*B) activation, and proinflammatory cytokine expression. In addition, berberine also inhibited apoptosis and diminished the cytochrome P450 (CYP) 2E1 expression in CSP-treated kidneys. CYP2E1 is the primary enzyme involved in the biotransformation of cisplatin, and previous studies have also demonstrated that genetic ablation of CYP2E1 imparted renoprotection against CSP-induce toxicity [27, 28].

### 2.6. Bixin

Bixin is the main carotenoid found in species of the tropical plant* Annatto* (*Bixa orellana*). Bixin inhibited CSP-induced nephrotoxicity by inhibiting lipid peroxidation and augmenting endogenous antioxidant defenses [29, 30]. However, further mechanistic studies are required to understand its renoprotective properties.

### 2.7. C-Phycocyanin (C-PC)

C-Phycocyanin (C-PC) is a pigment from the blue-green algae,* Spirulina maxima* [31]. C-PC has been shown to mitigate CSP-induced nephrotoxicity via inhibition of oxidative stress, inflammation, and apoptosis. Furthermore, mechanistic studies revealed that C-PC blunted CSP-induced proapoptotic mitogen-activated protein kinase (MAPK) kinase (MEK), B-cell lymphoma 2- (Bcl2-) associated X protein (Bax)/Bcl2 ratio alterations, and caspase-3 activation in renal tissues [31, 32].

### 2.8. Caffeic Acid Phenethyl Ester (CAPE)

Caffeic acid phenethyl ester (CAPE) is an active phenolic compound extracted from honeybee propolis [33]. CAPE treatment inhibited CSP-induced renal toxicity by suppressing oxidative stress, inflammation, and apoptosis. Further, CAPE also blunted CYP2E1 activation, thereby inhibiting the biotransformation of CSP [33, 34]. However, further studies are required to investigate whether CAPE provides renoprotection without compromising the anticancer effects of CSP.

### 2.9. Cannabidiol (CBD)

Cannabidiol (CBD) is a phenolic compound and phytocannabinoid extracted from the* Cannabis sativa* (marijuana) plant, and it elicits anti-inflammatory, immunomodulatory, and analgesic effects [35]. CBD attenuated CSP-induced nephrotoxicity by suppressing oxidative stress, inflammation, and apoptosis. It is also pertinent to note that CBD reversed the CSP-induced kidney injury when administered after the onset of renal tissue injury [36]. Furthermore, it is noteworthy that CBD is devoid of psychoactive properties since it does not bind to major cannabinoid receptors and has an excellent safety profile in human subjects. Recently, CBD was approved for the treatment of childhood epilepsy [25], and it could also be considered as a potent candidate for further development to counteract CSP-induced renal toxicity.

### 2.10. Capsaicin

Capsaicin is the major pungent ingredient in red peppers and has been used in pain sensation studies based on its stimulation of vanilloid receptor-1, an ion channel protein expressed by nociceptive primary afferent neurons [37]. Capsaicin has been demonstrated to inhibit oxidative stress, inflammation, and apoptosis in the renal tissues of CSP-treated animals. The renoprotective effects were in part due to the activation of heme oxygenase-1 (HO-1) [38, 39].

### 2.11. Cardamonin

Cardamonin is a flavone found in* Alpinia* plants and has been shown to affect cell-signaling pathways and to possess anticancer and anti-inflammatory properties [40]. Cardamonin increased endogenous antioxidants and decreased oxidative stress and inflammation [41–44].

### 2.12. Carnosic Acid

Carnosic acid is a naturally occurring polyphenolic diterpenoid molecule present in rosemary (*Rosmarinus officinalis*) [45]. Carnosic acid suppressed CSP-induced nephrotoxicity by mitigating oxidative stress and apoptosis in renal tissues [45]. However, additional studies are required to understand the molecular mechanisms purported to mediate its renoprotective actions.

### 2.13. Chrysin

Chrysin (5,7-dihydroxyflavone) is a flavonoid extracted from honeybee propolis. Chrysin has been reported to be a potent inhibitor of aromatase and anticancer properties [46]. Sultana et al. demonstrated that treatment of chrysin effectively diminished CSP-induced oxidative stress by improving antioxidant enzyme status and restored membrane integrity of tubular epithelial cells [47]. Furthermore, Khan et al. [48] reported that chrysin attenuated CSP-renal toxicity by inhibiting oxidative stress, p53 expression, DNA damage, and apoptosis.

### 2.14. Cinnamic Acid and Cinnamaldehyde

The essential oil of cinnamon contains both cinnamic acid (CA) and cinnamaldehyde (CD). These phytochemicals have been documented to possess antioxidant, antibacterial, and anti-inflammatory effects [49]. CA and CD administration to rodents restored kidney function, suppressed oxidative stress, and mitigated the histopathological degeneration induced by CSP [49]. However, additional studies are required to understand the precise molecular mechanism underlying the renoprotective actions of CA and CD.

### 2.15. Curcumin

Curcumin is a principle curcuminoid (phenolic terpene compound) derived from the Indian curry spice turmeric (*Curcuma longa*) [50]. Curcumin treatment restored CSP-induced depletion of endogenous antioxidants [51–53] and reduced inflammation by suppressing NF*κ*B activation, expression of proinflammatory cytokines, and adhesion molecules [54, 55]. Furthermore, curcumin has been reported to ameliorate CSP-induced renal toxicity by augmenting silent mating type information regulation 2 homolog-1 (SIRT-1) and nuclear factor erythroid-derived 2 (Nrf2), which enhanced endogenous antioxidant defenses and mitochondrial biogenesis [55, 56].

### 2.16. Cyanidin

Proanthocyanidins are polyphenol derivatives of flavan-3-ol flavonoids derived from grape seed. Proanthocyanidins are reported to possess antioxidant, anti-inflammatory, and antitumor activities [57]. Cyanidin treatment of rodents suppressed CSP-induced renal reactive oxygen species (ROS) generation and enhanced the activation of prosurvival kinases such as extracellular signal-regulated kinase (ERK) and Akt. Furthermore, cyanidin also suppressed CSP-induced renal apoptosis by blunting caspase-3/12 expression, the Bax/Bcl-2 ratio, p53 phosphorylation, and poly adenosine diphosphate (ADP) ribose polymerase (PARP) activation. In addition, cyanidin also suppressed CSP-induced endoplasmic reticulum stress in renal tissues [58]. Collectively these results suggest that cyanidin recruited several prosurvival pathways to counteract CSP-induced renal damage.

### 2.17. Decursin

Decursin is a natural pyranocoumarin compound isolated from the Korean herb* Angelica gigas* and is reported to possess anticancer activity [59]. Decursin treatment reduced CSP-induced renal toxicity by attenuating oxidative stress, inflammation, and apoptosis pathways in renal cancer cell lines and rodents [59, 60]. Recently, dose escalation studies were conducted to determine the pharmacokinetic profile of decursin in human subjects. From this study, it was inferred that decursin was well tolerated in both sexes and reached a peak plasma concentration in 8–12 h. These observations indicate the efficacy, safety, tissue distribution, and pharmacodynamic properties of decursin in human subjects [61].

### 2.18. Ellagic Acid

Ellagic acid is a naturally occurring phenolic compound found in fruits such as raspberries, strawberries, and pomegranates [62]. Ellagic acid treatment ameliorated CSP-induced renal toxicity by suppressing the kidney injury molecule (KIM-1) and clusterin protein expression (considered as early indicators of kidney injury) [63]. Furthermore, ellagic acid enhanced the glomerular filtration rate, which corroborated its reduction of inflammatory mediators and apoptotic markers in renal tissues [64]. These findings were correlated with the amelioration of CSP-induced tubular necrosis, degeneration, karyomegaly, and tubular dilatation [65].

### 2.19. Emodin

Emodin is the most abundant bioactive anthraquinone extracted from the Chinese culinary herb, Rhubarb (*Rheum palmatum*), and it possesses anticancer [66] and antioxidant activities [67]. Emodin treatment increased the cell viability after CSP treatment of normal human renal tubular epithelial cells [68]. In addition, emodin attenuated CSP-induced renal damage by suppressing the activity of* N*-acetyl-beta-D-glucosaminidase (NAG) [69], which is a lysosomal enzyme that is constitutively expressed in the proximal kidney tubule. Owing to its high molecular weight, under physiological conditions, NAG does not void via the kidneys because of its negligible glomerular filtration [70]. However, damage to the renal tubules causes the release of NAG in higher amounts than usual and, hence, it is excreted in the urine, and its serum accumulation is increased [70]. In a separate study, Liu et al. [71] demonstrated that emodin ameliorates CSP-induced apoptosis of rat renal tubular cells* in vitro* by modulating adenosine monophosphate-activated protein kinase (AMPK)/mechanistic target of rapamycin (mTOR) signaling pathways and activating autophagy and in vivo by suppressing caspase-3 activity and apoptosis in renal tissues.

### 2.20. Epigallocatechin-3-Gallate (EGCG)

Epigallocatechin-3-gallate (EGCG) is a phenolic compound present in green tea [72] and is an effective ROS scavenger* in vitro* and* in vivo* [73, 74]. EGCG mitigated CSP-induced nephrotoxicity by inducing the expression of* Nrf-2* and HO-1 and decreasing that of NF*κ*B and proinflammatory cytokines [72]. Furthermore, EGCG also inhibited endoplasmic reticulum (ER) stress-induced apoptosis through the suppression of phosphorylated (p)-ERK, glucose-regulated protein 78 (GRP78), and the caspase-12 pathway [75]. Furthermore, EGCG inhibited the ligand of death receptor Fas (Fas-L); apoptosis regulator, Bax; and the tumor-suppressor protein, p53, while it increased the expression of Bcl-2 and, thereby, inhibited the extrinsic pathways of renal cell apoptosis [76]. All these studies collectively established the renoprotective actions of EGCG.

### 2.21. Genistein

Genistein is a polyphenol nonsteroidal isoflavonoid phytoestrogen extracted from soybean. Genistein treatment counteracted CSP-induced ROS generation and suppressed NF*κ*B activation, proinflammatory cytokines expression, and apoptosis [77].

### 2.22. Ginsenosides Rh_4_ and Rk_3_

Ginseng is the root of* Panax ginseng* and is one of the most widely recommended and intensively studied herbal medicines. Ginsenosides are the secondary metabolites and unique constituents of* Panax* plants. Baek et al. [78] demonstrated that ginsenosides increased cell viability and prevented lactate dehydrogenase (LDH) leakage induced by CSP in normal renal proximal tubular epithelial cells. Furthermore, ginsenosides ameliorated CSP-induced renal damage by mitigating inflammation and apoptosis, which was evidenced by the suppression of DNA damage-induced apoptosis biomarkers such as phosphorylated c-Jun N-terminal kinase (JNK), p53, and cleaved caspase-3 expressions [79, 80].

### 2.23. Glycyrrhizic Acid

Glycyrrhizin and its aglycone glycyrrhetic acid (GA) are used for various therapeutic purposes in Chinese traditional medicine practice [81]. GA is the hydrophilic part of glycyrrhizin, an active compound found in licorice (*Glycyrrhiza glabra*), which is a conjugate of two molecules of glucuronic acid and GA. It is used as a flavoring agent in candies, pharmaceuticals, and tobacco products [82]. Furthermore, it has been reported to elicit anti-inflammatory, antioxidant, and antitumor activities [83]. GA treatment restored the antioxidant status and improved kidney function, as evidenced by diminished DNA fragmentation [82]. In addition, the renoprotective effects of GA were also associated with the upregulation of Nrf2 and downregulation of NF*κ*B expression, resulting in decreased kidney damage [84].

### 2.24. Hesperidin

Hesperidin is a pharmacologically active bioflavonoid found in citrus fruits [85]. Hesperidin attenuated CSP-induced renal toxicity by ameliorating oxidative stress, inflammation, and apoptosis [85, 86]. However, additional studies are required to understand the exact molecular mechanism mediating the renoprotection induced by hesperidin.

### 2.25. Isoliquiritigenin (ISL)

Isoliquiritigenin (ISL) is a flavonoid with a chalcone moiety extracted from several* Glycyrrhiza* species [87]. ISL has been shown to exert a variety of biological activities such as antiplatelet aggregation, antioxidant, and anti-inflammatory [88]. ISL exerted a remarkable renoprotective effect against CSP-induced renal toxicity by abrogating oxidative stress and apoptosis [87]. However, the precise molecular mechanisms purported to mediate the renoprotective activity of ISL needs to be explored.

### 2.26. Licochalcone A (LCA)

Licochalcone A (LCA) is a species-specific phenolic constituent of* Glycyrrhiza inflata*. LCA administration to CSP-treated animals restored kidney function markers and decreased oxidative stress [89]. However, the exact mechanism underlying the renoprotection induced by LCA needs to be investigated.

### 2.27. Ligustrazine

Ligustrazine (tetramethylpyrazine) is an alkaloid compound extracted from the Chinese herb Chuanxiong (*Ligusticum chuanxiong *Hort) [90] and is extensively used in China for the management of myocardial and cerebral infarction [91]. Ligustrazine significantly diminished CSP-induced urinary NAG excretion and renal tubular injury in a dose-dependent manner. Furthermore, ligustrazine also suppressed renal oxidative stress, inflammation, and apoptosis by restoring the Bax/Bcl-2 ratio [90].

### 2.28. Luteolin

Luteolin is a flavone present in high concentrations in celery, green pepper, and chamomile, and it has been reported to display anti-inflammatory, antioxidant, and anticarcinogenic activities [92, 93]. Luteolin treatment significantly reduced the pathophysiological changes induced by CSP in the kidneys by the suppression of oxidative/nitrative stress, inflammation, and apoptosis [92]. Moreover, luteolin also ameliorated tubular necrosis, which was confirmed using a terminal deoxynucleotidyl transferase (TdT) deoxyuridine 5′-triphosphate (dUTP) nick-end labeling (TUNEL) assay, and it diminished p53 activation and PUMA-*α* expression, as well as altering the Bax/Bcl-2 ratio [93].

### 2.29. Lycopene

Lycopene is a carotenoid pigment found in tomato [94]. Lycopene from dietary sources has been shown to reduce the risk of some chronic diseases including cancer and cardiovascular disorders [95]. The administration of lycopene significantly normalized the kidney function and antioxidant status of CSP-treated animals. Furthermore, lycopene also increased the expression of the organic anion and cation transporters (OAT and OCT, resp.) including OAT1, OAT3, OCT1, and OCT2 in the renal tissues [96–98]. In addition, lycopene also decreased the renal efflux transporters (multidrug resistance-associated protein [MRP]-2 and MRP4) levels and induced Nrf2 activation, which activated the antioxidant defense system [99]. Furthermore, lycopene protected against CSP-induced renal injury by modulating proapoptotic Bax and antiapoptotic Bcl-2 expressions and enhancing heat shock protein (HSP) expression [97].

### 2.30. Naringenin (NAR)

Citrus fruits (such as oranges and grapefruits) are rich in the flavanone naringenin (NAR, aglycone) [100]. NAR diminished the extent of CSP-induced nephrotoxicity by improving renal function and antioxidant enzyme activity and diminishing lipid peroxidation [101]. However, the detailed molecular mechanism of the renoprotective action of NAR against CSP-induced renal tissue injury is still unknown and requires further investigation.

### 2.31. Paeonol

Paeonol is a major phenolic component of Moutan cortex [102]. In traditional medicine practice, paeonol is used to treat various diseases including atherosclerosis, infections, and other chronic inflammatory disorders [103]. Paeonol improved kidney function and suppressed the levels of proinflammatory cytokines, which attenuated the renal tissue injury induced by CSP [102]. However, additional mechanistic studies are warranted to understand the renoprotective activity of paeonol.

### 2.32. 1,2,3,4,6-Penta-*O*-galloyl-*β*-D-glucose (PGG)

1,2,3,4,6-Penta-*O*-galloyl-*β*-D-glucose (PGG) is a polyphenol and water-soluble gallotannin isolated from the Chinese herb* Rhus chinensis* [104]. PGG significantly blocked cytotoxicity and reduced the sub-G1 accumulation of human renal proximal tubular epithelial cells induced by CSP [105]. In addition, PGG suppressed PARP cleavage, caspase-3 activation, cytochrome c release, and upregulation of Bax and p53 expression, which diminished apoptosis in the renal tissues [106].

### 2.33. Platycodin D (PD)

Triterpenoid saponins extracted from the roots of* Platycodon grandiflorum* exhibit a variety of pharmacological activities such as anti-inflammatory, anticancer, and immune-enhancing effects. The saponins in* P. grandiflorum* inhibited inducible nitric oxide synthase (iNOS) and cyclooxygenase-2 (COX-2) expressions by mitigating NF*κ*B activation in CSP-treated kidneys [107]. Furthermore, PD also ameliorated CSP-induced renal injury as revealed by the decreased intraluminal cast formation and diminished epithelial desquamation. These effects were mediated in part by quenching ROS generation and suppressing the apoptosis cascade [108].

### 2.34. Quercetin

Quercetin is one of the most abundant flavonoids found in several plant species and exerts numerous beneficial effects on health including cardioprotection, anti-inflammatory, anti-proliferative, and anticancer activities [109]. Quercetin ameliorated CSP-induced nephrotoxicity by mitigating oxidative stress, inflammation, and cell death pathways. Specifically, quercetin diminished renal lipid peroxidation, MAPK, and NF*κ*B activation, proinflammatory cytokine expression, and caspase activation, as well as decreasing apoptosis. The improvements in the molecular pathology induced by quercetin corroborated the improved renal function in CSP-treated animals [110–113].

### 2.35. Resveratrol

Resveratrol is a phenolic compound present in several botanical species such as mulberries, peanuts, red grapes, cranberries, and blueberries [114]. Resveratrol attenuated CSP-induced nephrotoxicity by augmenting the endogenous antioxidant defense system via SIRT1 and Nrf2 activation. Furthermore, it inhibited inflammatory cytokine production by blunting NF*κ*B activation and immune cell infiltration in renal tissues. In addition, resveratrol also inhibited CSP-induced renal apoptosis by downregulating p53 expression and restoring the Bax/Bcl-2 ratio. Furthermore, resveratrol enhanced the chemosensitivity of CSP without compromising its antitumor activity [115–118].

### 2.36. Rosmarinic Acid

Rosmarinic acid is an ester of caffeic acid that is abundantly present in rosemary (*Rosmarinus officinalis*) [119]. Rosmarinic acid treatment diminished the CSP-induced renal toxicity by attenuating oxidative stress, and this effect was characterized by decreased accumulation of 4-hydroxynonenal (4-HNE) formation with improvement in superoxide dismutase (SOD) activity and glutathione (GSH) levels. The beneficial effects of rosmarinic acid, in part, were mediated by its inhibition of the expression and activity of CYP2E1. In addition, rosmarinic acid inhibited CSP-induced inflammation by blunting NF*κ*B activation and apoptosis by reducing p53 activation and DNA damage [120].

### 2.37. Rutin

Rutin is a glycone of quercetin, which has been extracted from various citrus fruits [121]. The mechanism of the renoprotection induced by rutin against CSP toxicity is mediated by the suppression of oxidative stress, NF*κ*B activation, inflammatory cytokine expression, and apoptosis [86, 122].

### 2.38. Schizandrin and Schizandrin B

Schizandrin is a lignan found in the Chinese berry (*Schisandra chinensis*) [123]. Giridharan et al. [124] documented that schizandrin B inhibited CSP-induced oxidative stress, inflammation, and apoptosis by attenuating NF*κ*B, p53 accumulation, and cleaved caspase-3 expression. Furthermore, schizandrin B induced the activation of Nrf2 and its downstream target genes such as HO-1 and gamma-glutamylcysteine synthetase (*GGCS*), which is the rate-limiting enzyme involved in GSH synthesis. Furthermore, schizandrin B also inhibited CSP-induced nicotinamide adenine dinucleotide phosphate (NAD[P]H) dehydrogenase [quinone] 1 (NQO1) enzymatic activity. It is pertinent to note that NQO1 is involved in the one-electron reduction of quinones which produces superoxide and, thereby, propagates oxidative stress [125].

### 2.39. Silibinin

Silibinin is a flavonoid extracted from* Silybum marianum*, popularly known as the milk thistle [126]. Gaedeke et al. [127] demonstrated that silibinin inhibited CSP-renal damage by preserving the proximal tubular function and ameliorating proteinuria. However, the precise molecular mechanism underlying this action was not investigated. In another study, silibinin protected the kidneys against CSP-induced renal toxicity without compromising the antitumor activity of CSP in rodents [128].

### 2.40. Sulforaphane

Sulforaphane is an isothiocyanate present in cruciferous vegetables such as broccoli, Brussels sprout, and cabbage [129]. Sulforaphane inhibited CSP-induced renal dysfunction, structural damage, oxidative/nitrative stress, inflammation, and apoptosis. Mechanistically, sulforaphane attenuated MAPK and NF*κ*B activation and stimulated Nrf2 activation [130, 131]. In addition, several synthetic analogs of sulforaphane also exerted renoprotective activity against CSP-induced nephrotoxicity by the aforementioned mechanisms [132].

### 2.41. Tannic Acid

Tannins belong to the class of polyphenols and have been shown to possess multiple biological activities including anticancer [133], antioxidant, and antimicrobial activities [134]. Yokozawa et al. [135] demonstrated that tannic acid administration restored antioxidant levels, decreased lipid peroxidation, and improve renal function. Tannic acid also decreased CSP-induced DNA fragmentation by diminishing p53 activation [136]. Furthermore, green tea tannin has been reported to restore the kidney function and synergistically enhance the cell death of ovarian cancer cells by CSP [137]. In addition, Tikoo et al. [138] reported that tannic acid decreased PARP cleavage, phosphorylation of p38, and hypoacetylation of histone H4, which diminished kidney injury, indicating the efficacy of tannic acid as a therapeutic drug for CSP-induced nephrotoxicity.

### 2.42. Thymoquinone

Thymoquinone is a bioactive compound derived from* Nigella sativa* popularly known as black seed oil. Thymoquinone has been shown to exert anti-inflammatory, antioxidant, and antineoplastic effects in both* in vitro* and* in vivo* studies [139]. Thymoquinone was shown to improve kidney function, diminish lipid peroxidation, and augment endogenous antioxidants [139]. In addition, thymoquinone has also been shown to increase the expression of various organic anion and cation transporters such as OAT1, OAT3, OCT1, and OCT2, which are necessary for the renal clearance of xenobiotic agents including toxins and commonly used drugs [140, 141].

### 2.43. Xanthorrhizol

Xanthorrhizol is one of the major constituents from the rhizomes of* Curcuma xanthorrhiza,* a medicinal plant native to Indonesia [142]. Kim et al. [143] demonstrated the renoprotective action of xanthorrhizol against CSP-induced nephrotoxicity mediated by inhibiting NF*κ*B and activator protein-1 (AP-1) activation, proinflammatory cytokine expression, immune cell infiltration, and apoptosis. Furthermore, mechanistic studies revealed that xanthorrhizol suppressed CSP-induced phosphorylation of c-Jun N-terminal kinase (JNK) and p53, as well as the shutdown of the mitochondria-mediated apoptosis pathway [144].

### 2.44. Renoprotective Actions of Phytochemicals in Human Studies

The review of the published literature revealed that several preclinical studies reported the renoprotective properties of phytochemicals. Currently, there is no significant evidence from clinical trials indicating that phytochemicals show renoprotective efficacy in human subjects undergoing CSP chemotherapy. However, a recent open-labeled randomized clinical trial undertaken in a small patient population suggested that treatment with cystone (a herbomineral ayurvedic formulation) in combination with CSP chemotherapy improved renal function without compromising the antitumor effects of CSP. However, long-term follow-up data and survival rates were not presented in this study and, therefore, more stringent, well-designed, and controlled clinical trials are warranted to establish the clinical efficacy of cystone in combating CSP-induced nephrotoxicity [145].

## 3. Conclusion

The analysis of literature suggests that plant-derived agents (phytochemicals) are widely used to prevent the CSP-induced renal toxicity, and it is evident that these compounds exhibited potentially effective renal protection in preclinical studies. However, the major impediment to the clinical translation of these compounds for further pharmaceutical development pertains to the lack of convincing evidence of their bioavailability in human subjects [146, 147]. In addition, the therapeutic indexes for various phytochemicals are presently unknown. Therefore, future studies should investigate the analogs and derivatives of phytochemicals with demonstrable bioavailability in human subjects, and these molecules should be thoroughly investigated in preclinical models for further pharmaceutical development. In addition, most studies reported in the literature demonstrated the prophylactic action of phytochemicals in combating CSP-induced renal tissue injury. However, this approach has major limitations because clinically patients require treatment after and not before the onset of kidney damage. Therefore, future studies should essentially investigate the therapeutic effect of phytochemicals against CSP-induced nephrotoxicity in preclinical models. Specifically, studies must report the effect of phytochemical administration after the establishment of renal tissue injury and present the survival rate of the animal models. Finally, to establish the renoprotective actions of phytochemicals, studies need to be conducted in rodents harboring tumors that are sensitive to CSP. This is to ascertain that the beneficial effects of the phytochemicals do not compromise or interfere with the antitumor activity of CSP.

## Figures and Tables

**Table 1:** Phytochemicals investigated for renoprotective actions against cisplatin- (CSP-) induced nephrotoxicity.

Phytochemical	Dose, duration, and route of administration	Animal model	Cisplatin dose and route of administration	Key findings	Reference
NIF_1_ and 23-hydroxytormentic acid	10 mg/kg/day, orally (PO) for 14 days intraperitoneally (i.p.)	Sprague Dawley (SD) rats	7 mg/kg, i.p.	↓ BUN and serum creatinine ↓ MDA production and GSH depletion	[14]

6-Gingerol	12.5, 25, and 50 mg/kg for 5 days (before and after treatment), i.p.	Wistar rats	5 mg/kg, i.p.	↓ oxidative stress	[17]

6-Hydroxy-1-methylindole-3-acetonitrile	5 and 10 mg/kg, single dose, PO	LLC-PK1 cells and SD rats	7 mg/kg, i.p.	↓ BUN, creatinine, and urinary LDH ↑ HO-1 expression, activities of SOD, CAT, GR, MDA, and GSH	[19]

*β*-Caryophyllene	1–10 mg/kg, i.p. single dose	C57BL/6J mice	25 mg/kg, i.p.	↓ inflammation and dysfunction ↓ NOX-2 and NOX-4 expression, 4-HNE, 3-NT accumulation, and cell death	[23]

Berberine	1–3 mg/kg, single dose, i.p.	BALB/cN mice	13 mg/kg, i.p.	↓ BUN, creatinine, and oxidative/nitrosative stress ↓ NF-*κ*B, TNF-*α*, COX-2, iNOS, and histopathological changes ↓ p53 and active caspase-3	[27]

Bixin	2.5 and 5 mg/kg for 3 days, i.p.	Wistar rats	5 mg/kg, i.p.	↓ lipid peroxidation and renal glutathione depletion ↓ chromosome aberrations	[29, 30]

C-Phycocyanin	5–50 mg/kg, i.p.	C57BL/6J and CD1 mice	12–18 mg/kg, i.p.	↓ BUN, creatinine, oxidative stress, and apoptosis ↓ p-ERK, p-JNK, and p-p38 expression and Bax, caspase-9, and caspase-3 activation	[31, 32]

Caffeic acid phenethyl ester	10 *μ*M/kg, single dose, i.p.	Wistar Albino rats	7 mg/kg, i.p.	↓ BUN, tubular damage, and oxidative tissue damage ↑ antioxidant enzymes	[33]

Cannabidiol	2.5–10 mg/kg, i.p. (before and after treatment)	C57BL/6J mice	20 mg/kg, i.p.	↓ BUN, creatinine, ROS formation, and 3-NT ↓ PARP, caspase-3/7, and DNA fragmentation ↓ mRNA of TNF-*α* and IL1 and iNOS and protein expression	[36]

Capsaicin	5 and 10 mg/kg, PO for 6 days, i.p.	SD rats	5 mg/kg, i.p.	↓ BUN, creatinine, MDA, and renal damage	[38]
	2.5, 5, and 10 mg/kg for 5 days, i.p.	C57BL/6 mice	5 mg/kg, i.p.	↑ HO-1 expression	[39]

Cardamonin	10 and 30 mg/kg, PO for 2 weeks	Albino rats	7 mg/kg, i.p.	↑ SOD, GSH ↓ NOX-1, caspase-3 expression, and Bax/Bcl-2 ratio	[41]

Carnosic acid	100 mg/kg, PO for 10 days	Wistar rats	7.5 mg/kg, i.p.	↓ BUN, creatinine, and MDA ↑ GSH levels, catalase, SOD, GST, GPx, and GR activities ↓ caspase-3 activity, apoptosis, and renal damage	[45]

Chrysin	25 or 50 mg/kg 14 days, i.p.	Wistar rats	7.5 mg/kg, i.p.	↓ oxidative stress and apoptosis	[47]

Cinnamic acid (CA) and cinnamaldehyde (CD)	CA, 50 mg/kg CD, 40 mg/kg, PO for 7 days	SD rats	5 mg/kg, i.p.	↓ urea, creatinine, and MDA content ↑ GSH levels, SOD, CAT, and GPx activities	[49]

Curcumin	100 mg/kg 10 days, i.p.	Wistar rats	7 mg/kg, i.p.	↓ MDA ↑ NAMPT, SIRT1, SIRT3, and SIRT4 levels	[56]
	100 mg/kg, i.p.	C57BL/6J mice	20 mg/kg, i.p.	↓ renal TNF-*α*, MCP-1, and ICAM-1 mRNA expression	[54]
	8 mg/kg	Wistar rats	5 mg/kg, i.p.	↓ creatinine, TBARS, and MDA	[51]

Cyanidin	10, 20, and 40 *µ*g/mL	HK-2 cells	8 *µ*g/mL	↓ BUN, creatinine, MDA, renal index, and IL-6 ↓ GRP78, p-ERK, caspase-12, and PARP cleavage ↓ apoptosis, DNA damage, ERK activation, and AKT inhibition	[58]

Decursin	20–80 mM	Primary HRCs	20–80 mM	↑ catalase, SOD, and GPx activities ↓ caspases 3 and 9, PARP, DNA fragmentation, and apoptosis	[59]
	10–40 mg/kg 3 days, i.p.	SD rats	5.2 mg/kg, i.p.	↓ BUN and creatinine	[60]

Ellagic acid	10 and 30 mg/kg 9 days, i.p.	SD rats	6 mg/kg, i.p.	↓ creatinine, urea, and kidney injury ↑ total antioxidant status and GSH	[63]
	10 mg/kg 10 days, i.p.	SD rats	7 mg/kg, i.p.	↓ MDA levels and improved antioxidant enzymes ↓ tubular necrosis and tubular dilatation	[65]

Emodin	10 mg/kg for 9 days, i.p.	Wistar rats	6 mg/kg, i.p.	↑ GSH, TAC, GST, GPx, GR, SOD, and CAT ↓ NAG, creatinine, and urea concentration	[69]

Epigallocatechin-3-gallate	l00 mg orally, 2 days	Wistar rats	7 mg/kg, i.p.	↑ SOD, CAT, GPx, and GSH ↓ NF-*κ*B and 4HNE	[72]
	100 mg/kg i.p., single dose	C57BL/6 mice	20 mg/kg, i.p.	↓ p-ERK, GRP78, caspase-12, Fas-L, BAX, and apoptosis	[75, 76]

Genistein	10 mg/kg 3 days 25 *µ*g/L	C57BL/6 mice HK-2 cells	20 mg/kg, i.p. 1 *µ*g/mL	↓ BUN, creatinine, ROS production, tubular damage, and necrosis score ↓ ICAM-1 and MCP-1 expression and NF-*κ*B activation ↓ apoptosis and p53 induction	[77]

Ginsenosides	10–60 *µ*g/mL	LLC-PK1 cells	25 and 500 *µ*M	↓ LDH leakage, renal damage, and apoptosis	[78–80]

Glycyrrhizic acid	75 and 150 mg/kg for 7 days, i.p.	BALB/c and Swiss Albino mice	7 mg/kg, i.p.	↑ GSH, GR, GST, catalase, and GPx activities ↓ BUN and creatinine	[82, 84]

Hesperidin	100 and 200 mg/kg 10 days, i.p.	Wistar rats	7.5 mg/kg, i.p.	↓ BUN, creatinine, and DNA degradation ↑ SOD, GPx, GST, GR, GSH, and catalase activities and vitamin C levels ↓ renal TNF-*α* levels	[85, 86]

Isoliquiritigenin	1 mg/kg for 15 days, i.p.	BALB/c mice	5 mg/kg, i.p.	↓ BUN, creatinine, nitrite, and tissue MDA and ROS	[87]

Licochalcone A	1 mg/kg for 15 days, i.p.	BALB/c mice	5 mg/kg, i.p.	↓ BUN, creatinine, nitrite, and MDA	[89]

Ligustrazine	50 and 100 mg/kg, 7 days, i.p.	SD rats	8 mg/kg, i.p.	↓ urinary protein excretion, NAG excretion, creatinine, and BUN ↑ GSH levels, SOD, and GST activities ↓ tubular cell apoptosis	[90]

Luteolin	10 mg/kg 3 days, i.p.	BALB/cN mice	10 and 20 mg/kg, i.p.	↓ renal dysfunction, tubular injury, oxidative stress, BUN, and creatinine ↑ GSH, SOD, and catalase ↓ p53 activation and PUMA-*α* protein expression	[92]
	50 mg/kg 3 days, i.p.	C57BL/6J mice	20 mg/kg, i.p.	↓ CYP2E1, Bcl-2, 4-HNE, 3-NT, NF-*κ*B, and caspase-3 ↓ MRP4 and MRP2 expression	[93]

Lycopene	6 mg/kg 10 days, i.p.	Wistar rats	7 mg/kg, i.p.	↓ urea and creatinine and MRP2 and MRP4 expression ↑ OAT1, OAT3, OCT1, OCT2, Nrf2, and Bcl-2 expression	[96, 97]
	4 mg/kg 5 days, i.p.	SD and Wistar rats		↑ catalase, GPx, and SOD activities ↓ NF-*κ*B, HSP 60 and HSP 70, and Bax expression	[98]

Naringenin	20 mg · kg^−1^ · day^−1^, PO for 10 days	Wistar Albino rats	7 mg/kg, intravenous (i.v.)	↓ urea, creatinine, sodium excretion, and renal lipid peroxides ↑ GST activity and renal antioxidant enzymes	[101]

Paeonol	20 mg/kg 3 days, i.p.	BALB/c mice	10–30 mg/kg I.P.	↓ creatinine, BUN, TNF-*α*, and IL-1*β*	[102]

Penta-*O*-galloyl-*β*-D-glucose	20–80 *µ*M	Primary HRC	40 *µ*M	↓ cytotoxicity, apoptosis, PARP cleavage, Bax, and caspase-3 ↓ cytochrome C translocation and ROS production	[106]

Platycodin D	0.1, 1, and 5 mg/kg for 3 days, i.p.	ICR mice	20 mg/kg, i.p.	↓ BUN, creatinine, TBARS, NF-*κ*B activation, ↑ GSH, GPx, and SOD	[108]

Quercetin	100 mg/kg 30 days	Albino rats	12 mg/kg i.p.	↑ GSH, GPX, SOD, CAT, GR, XO, TOS, and TAC ↓ BUN, creatinine, LPO, H_2_O_2_, and tubular cell necrosis	[110]
	50 mg/kg 3 days	Wistar rats	5 mg/kg, i.p.	↓ Na and K excretion, NAG, LDH, ALP, GGT, and KIM-1 ↓ GSH/GSSG ratio, NF*κ*B, iNOS, ICAM-1, VCAM-1, and renal MPO	[111]
	50 and 100 mg/kg 9 days, i.p.	Fischer-F344 rats	7.5 mg/kg, i.p.	↓ caspase-3/7 activity and DNA fragmentation	[148]

Resveratrol	25 mg/kg single dose, i.p.	Albino mice	5 mg/kg, i.p.	↓ creatinine, MDA, and LDH leakage	[115]
	10 mg/kg, 7 days	C57BL/6 mice and	20 mg/kg, i.p.	↓ inflammation and necrosis	[116]
	30 *μ*g/mL, i.p.	Fischer rat kidney *in vitro*	7.5/15 *μ*g/mL, i.p.	↓ acetylation of p53 and SIRT1	[117]

Rosmarinic acid	1, 2, and 5 mg/kg 2 days, i.p.	BALB/cN mice	13 mg/kg, i.p.	↓ creatinine and BUN ↓ CYP2E1, HO-1, and 4-HNE expression ↓ NF*κ*B and cleaved caspase-3 expression	[120]

Rutin	75 and 150 mg/kg 21 days	Wistar rats	7 mg/kg, i.p.	↓ BUN, creatinine, H_2_O_2_, LDH, caspase-3, NF*κ*B, and TNF-*α* level	[122]
	30 mg/kg 14 days	SD rats	5 mg/kg, i.p.	↑ membrane integrity, GSH, XO, and GGT	[86]

Schizandrin and schizandrin B	10, 25, 50 mg/kg 15 days, i.p.	BALB/c mice	10 mg/kg, i.p.	↓ NF*κ*B activation and p53 activation	[124]

Silibinin	200 mg/kg single dose, i.p.	Wistar rats	5 mg/kg, i.p.	↑ creatinine clearance ↑ glomerular and proximal tubular function	[127, 128]

Sulforaphane	500 *μ*g/kg/day i.v. for 3 days	Wistar rats	7.5 mg/kg, i.p.	↓ p38 MAPK and renal adhesion molecule expressions	[130]
	500 *μ*g/kg/day i.p. for 3 days	Wistar rats	10 mg/kg, i.p.	↓ inflammatory cell infiltration	[131]

Tannic acid	40 and 80 mg/kg 7 days, i.p.	Swiss Albino mice	7 mg/kg, i.p.	↓ BUN, creatinine, p38 MAPK phosphorylation, and PARP cleavage ↓ XOR and LPO; ↑ G6PD, QR, and catalase activities	[136]

Thymoquinone	50 mg/L in drinking water for 5 days	Wistar Albino rats and Swiss Albino mice	5, 7, and 14 mg/kg i.v. in rats i.p. in mice	↓ urea, creatinine, MDA, 8-isoprostane, MRP2, and MRP4 ↑ OAT1, OAT3, OCT1, and OCT2 and survival rate of animals	[139]

Xanthorrhizol	100 and 200 mg/kg for 4 days, i.p.	ICR mice	45 mg/kg, i.p.	↓ BUN, creatinine, and lipid peroxides	[143]

**Table 2:** Structures of phytochemicals investigated for renoprotective action against cisplatin- (CSP-) induced nephrotoxicity.

Phytochemical	Structure	Chemical class
23-Hydroxytormentic acid	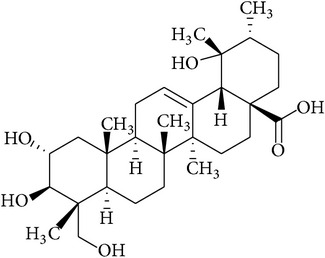	Carboxylic acid

6-Gingerol	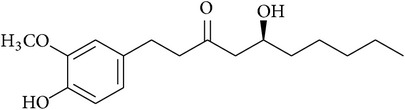	Decanone

6-Hydroxy-1-methylindole-3-acetonitrile	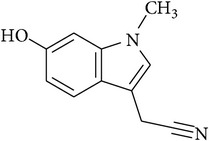	Nitrile

Caffeic acid phenylethyl ester	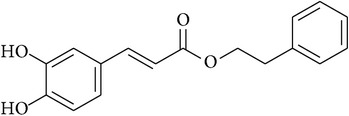	Ester

Cannabidiol	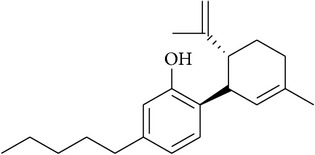	Monoterpene

*β*-Caryophyllene	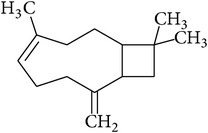	Bicyclic alkene

Cinnamaldehyde	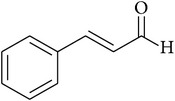	Aldehyde

Curcumin	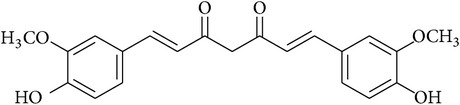	Diketone

Berberine	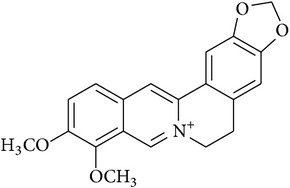	Isoquinoline

Bixin	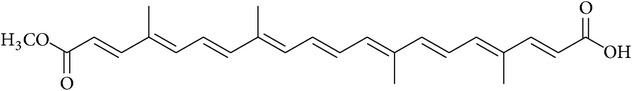	Apocarotenoid

C-Phycocyanin	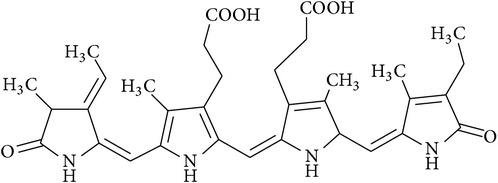	Phycobiliprotein

Capsaicin	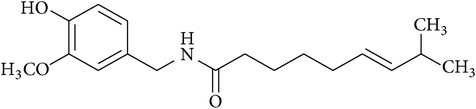	Amide

Cardamonin	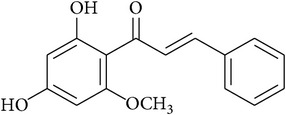	Chalconoid

Carnosic acid	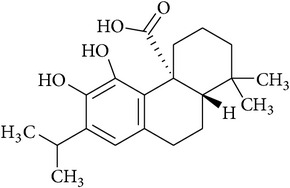	Benzenediol abietane diterpene

Chrysin	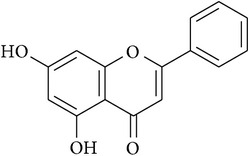	Flavonoid

Cinnamic acid	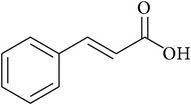	Carboxylic acid

Cyanidin	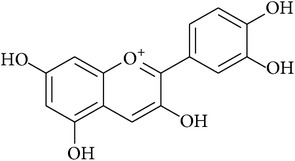	Anthocyanidin

Decursin	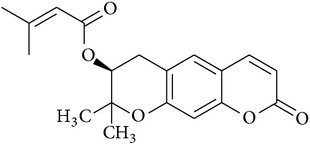	Coumarin

Ellagic acid	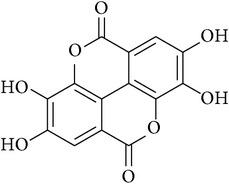	Chromene-5,10-dione

Emodin	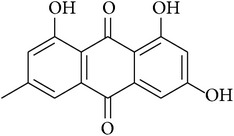	Anthraquinone

Epigallocatechin-3-gallate	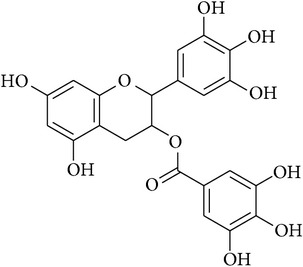	Polyphenol

Genistein	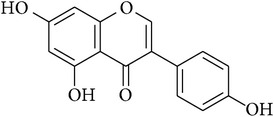	Isoflavone

Ginsenoside	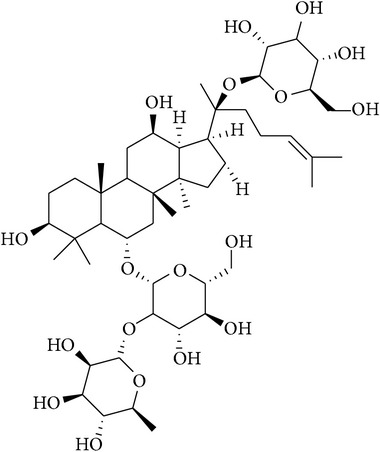	Triterpene-saponin

Glycyrrhizic acid	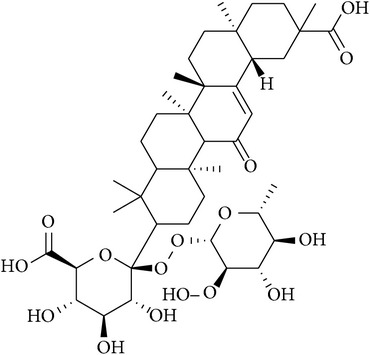	Triterpenoid saponin

Hesperidin	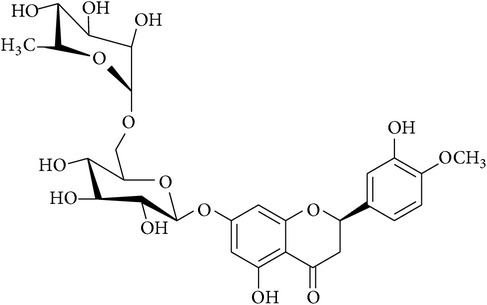	Licorice chalconoid

Isoliquiritigenin	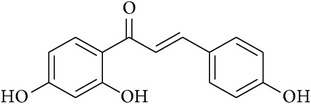	Chalconoid

Licochalcone A	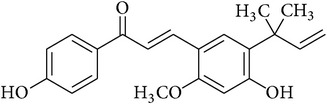	Chalconoid

Ligustrazine	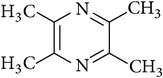	Pyrazine

Luteolin	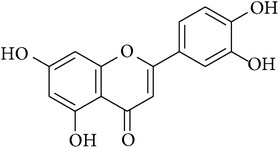	Flavanone

Lycopene	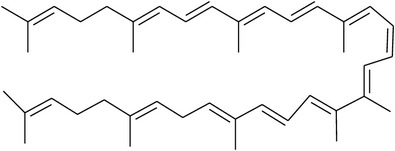	Carotenoid

Naringenin	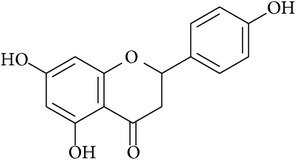	Flavanone

Paeonol	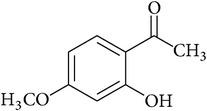	Acetophenone

Penta-O-galloyl-B-D-glucose	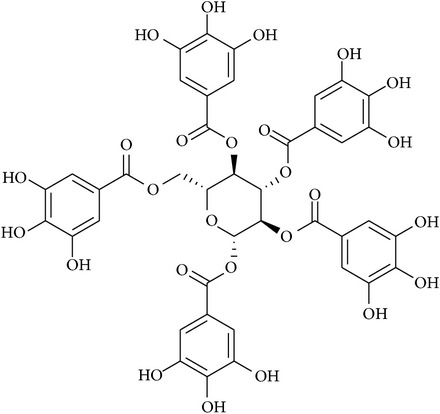	Glycoside

Platycodin D	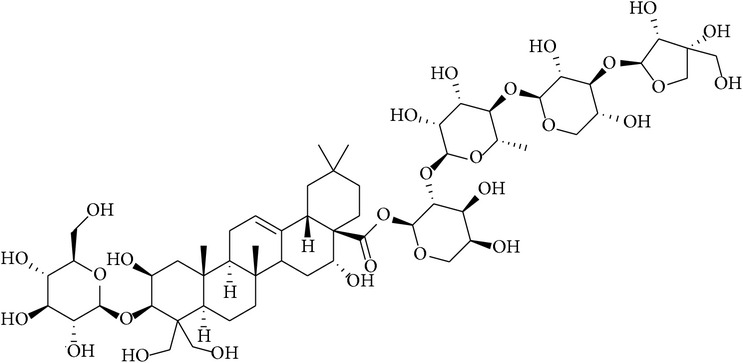	Saponin

Quercetin	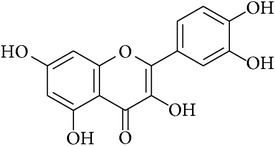	Flavonol

Resveratrol	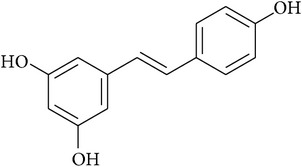	Stilbenoid

Rosmarinic acid	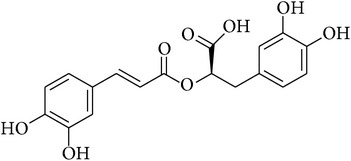	Caffeic acid

Rutin	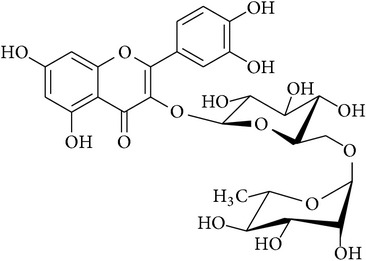	Chroman-4-one

Schizandrin	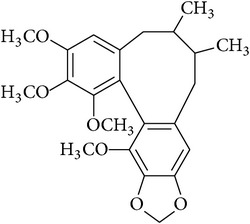	Cycloocta[1′,2′:4,5]benzo[1,2-d][1,3]dioxole

Silibinin	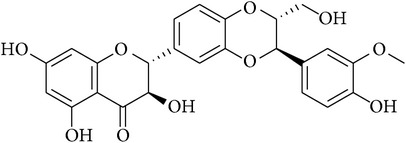	Chroman-4-one

Sulforaphane	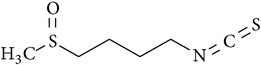	Isothiocyanate

Tannic acid	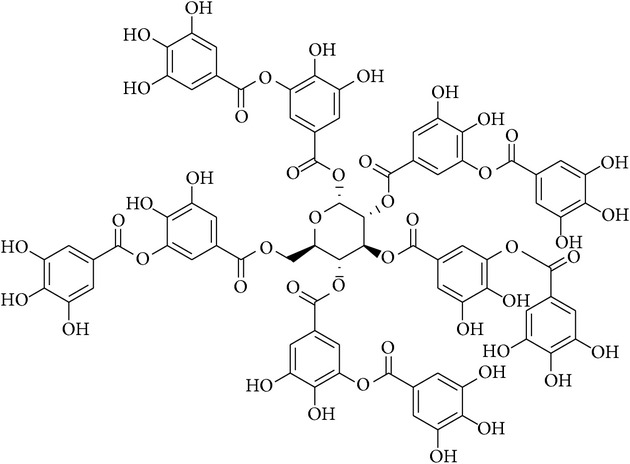	Polyphenol

Thymoquinone	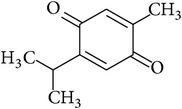	1,4-Quinone

Xanthorrhizol	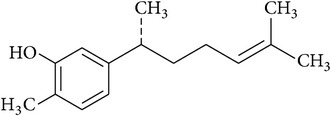	Sesquiterpene

## Abbreviations

CSP: Cisplatin
CB_2_: Cannabinoid receptor-2
NF-*κ*B: Nuclear factor kappa-light-chain-enhancer of activated B-cells
CYP2E1: Cytochrome P450 2E1
MAPK: Mitogen-activated protein kinase
Bax: B-cell lymphoma 2- (Bcl-2-) associated X protein
Bcl-2: B-cell lymphoma 2
HO-1: Heme oxygenase-1
p53: Tumor-suppressor protein p53
SIRT, Sirtuin: Silent mating type information regulation 2 homolog
Nrf2: Nuclear factor erythroid-derived 2
ROS: Reactive oxygen species
ERK: Extracellular signal-regulated kinases
Akt: Serine/threonine-specific protein kinase (synonym: protein kinase B)
PARP: Poly adenosine diphosphate (ADP) ribose polymerase
KIM-1: Kidney injury molecule-1
NAG: * N*-acetyl-D-glucosamine
AMPK: Adenosine monophosphate-activated protein kinase
mTOR: Mechanistic target of rapamycin
GRP-78: 78 kDa glucose-regulated protein
Fas-L: Fas ligand [synonym: cluster of differentiation antigen 95 (CD95) ligand]
LDH: Lactate dehydrogenase
JNK: c-Jun N-terminal kinases
TUNEL: Terminal deoxynucleotidyl transferase deoxyuridine 5′-triphosphate (dUTP) nick-end labeling
PUMA: p53-upregulated modulator of apoptosis
OAT: Organic anion transporter
OCT: Organic cation transporter
MRP: Multidrug resistance-associated proteins
HSP: Heat shock protein
iNOS: Inducible nitric oxide synthase
COX-2: Cyclooxygenase-2
4-HNE: 4-Hydroxynonenal
SOD: Superoxide dismutase
GSH: Glutathione (reduced)
GCLC: Gamma-glutamyl cysteine synthetase
NQO1: NAD(P)H dehydrogenase [quinone] 1
AP-1: Activator protein-1
BUN: Blood urea nitrogen
MDA: Malondialdehyde
CAT: Catalase
GR: Glutathione reductase
NOX: NADPH (nicotinamide adenine dinucleotide phosphate-oxidase) oxidase
3-NT/3-NY: 3-Nitrotyrosine
TNF-*α*: Tumor necrosis factor alpha
GPx: Glutathione peroxidase
p38: p38 MAPK
NO: Nitric oxide
IL-1*β*: Interleukin 1 beta
NAMPT: Nicotinamide phosphoribosyl transferase
MCP-1: Monocyte chemoattractant protein-1
ICAM-1: Intercellular adhesion molecule-1
TBARS: Thiobarbituric acid reactive substances
IL: Interleukin
TAC: Total antioxidant capacity
GST: Glutathione S-transferase
XO: Xanthine oxidase
TOS: Total oxidant status
LPO: Lipid peroxidation
H_2_O_2_: Hydrogen peroxide
ALP: Alkaline Phosphatase
GSSG: Glutathione disulfide
ICAM: Intercellular adhesion molecule
VCAM: Vascular cell adhesion protein
MPO: Myeloperoxidase
G6PD: Glucose-6-phosphate dehydrogenase
QR: Quinone reductase
I.P.: Intraperitoneal injection
I.V.: Intravenous administration
LLC-PK1: Renal epithelial cells derived from normal pig kidney
HK-2 cells: Proximal tubular epithelial cells from normal human kidney
HRCs: Human primary epithelial cells from cortex and glomeruli
ETC: Electron transport chain
XOR: Xanthine oxidoreductase
CXCL1: Chemokine (C-X-C motif) ligand 1
ER: Endoplasmic reticulum
PMN: Polymorphonuclear (neutrophil).
